# Editorial: Cross-Modal Learning: Adaptivity, Prediction and Interaction

**DOI:** 10.3389/fnbot.2022.889911

**Published:** 2022-04-04

**Authors:** Jianwei Zhang, Stefan Wermter, Fuchun Sun, Changshui Zhang, Andreas K. Engel, Brigitte Röder, Xiaolan Fu, Gui Xue

**Affiliations:** ^1^Department of Informatics, University of Hamburg, Hamburg, Germany; ^2^Department of Computer Science and Technology, Tsinghua University, Beijing, China; ^3^Department of Automation, Tsinghua University, Beijing, China; ^4^Department of Neurophysiology and Pathophysiology, University Medical Center Hamburg-Eppendorf, Hamburg, Germany; ^5^Department of Psychology, University of Hamburg, Hamburg, Germany; ^6^Institute of Psychology, Chinese Academy of Sciences, Beijing, China; ^7^Department of Psychology, University of Chinese Academy of Sciences, Beijing, China; ^8^State Key Laboratory of Cognitive Neuroscience and Learning & IDG/McGovern Institute for Brain Research, Beijing Normal University, Beijing, China

**Keywords:** crossmodal learning, multisensory processing, crossmodal integration, adaptivity, prediction

Crossmodal learning has in recent years emerged as a new area of interdisciplinary research. The term *crossmodal learning* refers to the synergistic synthesis of information from multiple sensory modalities such that the learning that occurs within any individual sensory modality can be enhanced with information from one or more other modalities. Crossmodal learning is a crucial component of adaptive behavior in a continuously changing world, and examples are ubiquitous, such as: learning to grasp and manipulate objects; learning to walk; learning to read and write; learning to understand language and its referents; etc. In all these examples, visual, auditory, somatosensory or other modalities have to be integrated, and learning must be crossmodal. In fact, the broad range of acquired human skills are crossmodal, and many of the most advanced human capabilities, such as those involved in social cognition, require learning from the richest combinations of crossmodal information. In contrast, even the very best systems in Artificial Intelligence (AI) and robotics have taken only tiny steps in this direction. Building a system that composes a global perspective from multiple distinct sources, types of data, and sensory modalities is a grand challenge of AI, yet it is specific enough that it can be studied quite rigorously and in such detail that the prospect for deep insights into these mechanisms is quite plausible in the near term. Crossmodal learning is a broad, interdisciplinary topic that has not yet coalesced into a single, unified field. Instead, there are many separate fields, each tackling the concerns of crossmodal learning from its own perspective, with currently little overlap. By focusing on crossmodal learning, this Research Topic brings together recent studies demonstrating avenues of progress in artificial intelligence, robotics, psychology and neuroscience.

Several articles of this Research Topic review recent developments in this emerging field and, thus, are well-suited to provide the reader with an overview and with a compact introduction to several aspects of particular interest. The review by Bruns focuses on ventriloquism, one of the classic examples of crossmodal integration and learning. The article provides an overview of established experimental paradigms to measure the ventriloquism effect and aftereffect and summarizes new results regarding the role of top-down influences, recalibration processes and brain networks involved in multisensory learning. Li et al. discuss applications of probabilistic models in machine signal processing and human psychophysics. Focusing on audio-visual processing, they aim to identify commonalities between probabilistic models addressing brain processes and those aiming at building intelligent machines. Fu et al. review studies of selective attention in unimodal and crossmodal settings from the perspectives of psychology and cognitive neuroscience, and evaluate different ways to implement analogous mechanisms in computational models and robotics. Alaçam et al. review the interaction of language and vision in human crossmodal processing and describe performance characteristics that facilitate the robustness of language understanding. Furthermore, they discuss how such empirical findings in humans can be applied for situated language comprehension in artificial systems. Focusing on neural mechanisms, Xu et al. review the interdependence of low- and high-level cortical areas for the emergence of crossmodal processing during development. Furthermore, they discuss the applicability and relevance of insights on biological crossmodal processing for brain-inspired intelligent robotics.

## Crossmodal Learning in Natural Systems

### Crossmodal Recalibration

Three articles of this Research Topic address the question of how vision calibrates representations of other sensory modalities, either audition (Ahmad et al.; Kramer et al.) or haptics (Katzakis et al.). Ahmad et al. investigated patients who have lost central vision due to a retinal disease called macular degeneration. They observe crossmodal changes, that is, not only visual but also additionally distorted auditory spatial representations. These results suggest that vision shapes auditory space. Kramer et al. experimentally investigate a similar question. It has been known for a long time that in case of discrepant auditory spatial stimulation, auditory localization is shifted toward the visual stimulus (the ventriloquist effect). After a repeated exposure to audio-visual spatial discrepant stimulation, auditory localization is adapted (the ventriloquist after effect), similar as shown over a longer time scale by Ahmad et al. in patients with macular degeneration. Kramer et al. provide evidence that both audio-visual integration and visual recalibration of auditory spatial are subject to top-down modulation rather than being exclusively bottom-up driven. Katzakis et al. demonstrate that the life-long ability of crossmodal recalibration allows human observes to adapt to virtual reality. They asked subjects to judge haptic size in the context of discrepant visual information in virtual reality and observed a similar visual dominance as known for real world situations.

### High-Level Cognitive Processes

It remains unclear how crossmodal information is integrated and represented in crossmodal learning. Three articles address this question in different ways. Using a congruency evaluation task, Spilcke-Liss et al. find that participants made more errors and responded more slowly to paired audio-visual stimuli accompanying with an unattended incongruent stimulus than with an unattended congruent stimulus. The results indicate that semantic incongruencies of crossmodal integration could occur even when they are not endogenously attended. Using a mental rotation task of digitally-rendered haptic objects, Tivadar et al. observe a typical mental rotation effect for trained letters. The findings indicate that multiple sensory modalities can support spatial computations and have important implications on how to mitigate visual impairments. Using a prototype category learning task, Zhou et al. find that participants could incidentally combine the sound and the defined visual features to form category knowledge. Moreover, a larger learning effect for the edge- than the surface-based category in implicit knowledge rather than explicit knowledge indicates that edge-based features play a more crucial role than surface-based features in implicit category learning.

### Mechanisms of Crossmodal Processing

Two of the articles in this Research Topic deal with mechanisms that may be involved in crossmodal learning. The study by Li et al. investigates the long-term dynamics of cortical activity patterns during the formation of multimodal memories by two-photon imaging of immediate early gene expression in the mouse. The results demonstrate that, in superficial cortical layers, the patterns show similar dynamics across structurally and functionally distinct cortical areas and can be consistent across several days. By contrast, in deep layers, the activity dynamics varies across different areas and is sensitive to activities at previous time points. These results suggest different roles of superficial and deep layer neurons in the long-term multimodal representation of the environment. A modeling study by Maye et al. investigates the learning of sequences of uni- and multisensory events which are presented in a rhythmic manner. The paper introduces a neurobiologically plausible computational model that captures the sequences by attuning an ensemble of neural oscillators. The learning properties of the model are compared with behavioral results from a study in human participants, yielding good agreement for sequences with different levels of complexity.

## Crossmodal Learning in Artificial Systems

### Sensorimotor Processing

Three articles in this Research Topic consider crossmodal learning of crossmodal perception and visuo-motor skills in robots. Unsupervised learning of multisensory bindings of visual and auditory stimuli is addressed by Barros et al. For example, humans quickly learn to associate a barking sound with the visual appearance of a dog, and continuously fine-tune this association over time. The authors develop a computational model for this task that addresses the important properties of expectation learning, namely the lack of explicit external supervision other than temporal co-occurrence. The proposed hybrid neural model is based on audio-visual autoencoders and a recurrent self-organizing network. The authors demonstrate the learning of concept bindings by evaluating the trained system on unisensory classification tasks on a large video corpus. Deng et al. introduce a grasp planning system that combines a computational visual attention model to locate regions of interest in a table scene with a deep convolutional neural network to predict grasp type and grasp contact areas. The system is trained on images of common household objects, each annotated with grasp type and finger contact regions. The approach is evaluated in simulation and real-world experiments, showing a speed-up and improved grasp stability over the tested baseline. The paper by Kerzel et al. introduces the NICO robot, a child-sized humanoid specially designed for both social interaction and manipulation experiments. To engage in social interaction, the robot can express stylized facial expressions and utter speech via an Embodied Dialogue System. The ability for social interaction is considered a key factor for companion robots that learn with the help of non-expert teachers, as these robots are capable of asking questions that are vital to their learning process. In the presented study, NICO acquires visuomotor grasping skills by interacting with its environment and human teachers with little or no prior experience with robots.

### Language Processing Grounded in Robotic Actions

In crossmodal language learning, information from multiple modalities is processed to form abstract semantic representations that are associated with language. Language itself can be regarded as an abstract modality that can be transferred differently, e.g., by acoustics, sign language or text. Three of the papers in this Research Topic (Heinrich et al.; Mi, Liang, et al.; Mi, Lyu, et al.) propose models to investigate the problem of language grounding in the context of adaptive and interacting robots. Heinrich et al. study early language learning in a neurocognitively plausible end-to-end model. While the robot interacts with the environment receiving language labels, the model neurons act on multiple timescales to self-organize hierarchically and capture abstract information. Mi, Liang, et al. apply affordance detection on the image objects and extract the semantic intention from the command, in order to predict abstract desires, such as “I am thirsty,” which do not refer to objects explicitly. Mi, Lyu, et al. use visual language grounding to address ambiguities of natural language queries in human-robot interactions. A referring expression comprehension network understands visual semantics while a scene graph network allows finding relevant regions on the image even when the given language commands are complex. These three related papers on language grounding include validations on complementary robots such as the humanoid NICO, the UR5 arm, and the Robotiq 3-finger gripper.

### Knowledge and Reasoning

Visual reasoning is a multimodal task that extends visual classification by requiring both abilities of comprehension and reasoning. Three papers in this Research Topic report results on visual reasoning in multiple domains, namely visual question answering (Su et al.), video captioning (Chen et al.), and knowledge graph generation (Mao et al.). Su et al. improve the state-of-the-art of visual reasoning in visual question answering. They extend a neural module network, which is capable of spatial reasoning over the input image, by a layout generation network, which learns a policy that combines primitive modules of reasoning. The policy is rewarded in a dual-image task and, as a result, generates more comprehensible reasoning steps than previous models. Chen et al. introduce multiple innovations to video captioning models. They improve the visual input features for better detection of semantics with adequate complexity, overcome some constraints of teacher-forcing by adding self-teaching, and propose a sentence-length-modulated loss function that promotes the model to generate longer, more expressive sentences. Mao et al. generate structured knowledge graphs from either text or images as inputs. To generate a semantic graph of a scene, a hybrid relation extractor iteratively predicts relation pairs with the use of explanatory logic rules. The model performs particularly well for dense knowledge graphs. Together, these three models demonstrate how state-of-the-art models can acquire knowledge and perform reasoning on large-scale real-world visual data.

## Outlook

Combined, the 22 papers in this Research Topic present an up-to-date and representative overview of current trends in the emerging research field of crossmodal learning, integrating contributions from psychology, neuroscience, artificial intelligence, and robotics. On the theory side, the review and survey papers collected in this volume all agree on the fundamental importance of Bayesian approaches for crossmodal information integration and learning. The computational models developed for the behavioral studies included here are based on this, and future research can be expected to follow this line as well. Except for one paper still reporting an elegant analytical model, the different application studies all propose deep neural networks trained on custom datasets, confirming the recent near-absolute dominance of deep learning approaches for complex artificial intelligence or robotics tasks. However, the proposed deep networks are all different and highly optimized toward their respective domain and input modalities. This remains in striking contrast to the operation of the mammalian brain, with its apparent ease to process, integrate, and memorize information from a variety of sensory channels using a surprisingly uniform structure. Proponents of deep learning often conjecture that performance will scale with network and training set size. We expect that the trend toward more complex networks trained on ever larger and more diverse multimodal datasets will continue, resulting in better AI applications as well as better computational models for neuroscience and psychology.

## Author Contributions

All authors contributed to the writing of this editorial. All authors approved the submitted version.

## Funding

This work was funded by the Sino-German Collaborative Research Centre “Crossmodal Learning” (DFG SFB TRR 169/NSFC 62061136001).

## Conflict of Interest

The authors declare that the research was conducted in the absence of any commercial or financial relationships that could be construed as a potential conflict of interest.

## Publisher's Note

All claims expressed in this article are solely those of the authors and do not necessarily represent those of their affiliated organizations, or those of the publisher, the editors and the reviewers. Any product that may be evaluated in this article, or claim that may be made by its manufacturer, is not guaranteed or endorsed by the publisher.

